# SASP: roadblock for tissue re-organization

**DOI:** 10.18632/aging.100602

**Published:** 2013-09-25

**Authors:** Julie Cahu

**Affiliations:** MILPAT (EA 4652), Faculté de Médecine, CHU, Caen, France

Senescence has been shown to prevent and promote tumorigenesis. [1, 2] These results are not so paradoxical.

To develop and maintain, organisms rely on cellular growth and cell division. Each of these processes is spatiotemporally finely tuned and both are tightly coordinated to ensure organism homeostasis throughout life. As organism aged, deregulations of these processes appear leading to hyperplastic or degenerating diseases, such as cancer and Alzheimer disease, respectively [3]. Interestingly, these two aged-related diseases have been linked to a cellular response that yet, uncouples cellular growth from cell division: senescence. Senescence is a natural cellular response that can be triggered by various stimuli, such as telomere shortening, oncogenic stresses or unrepaired DNA damages [4]. Senescent cells grow but do not divide so that they are enlarged and restricted in number. In addition, as they do not proliferate due to the irreversible cell cycle arrest, they do not differentiate. Thus senescence modifies tissue homeostasis by profoundly impacting tissue architecture both physically and biologically. Such disorganisation leads to alteration of cell contacts thereby re-wiring cellular communication. To communicate, cells use physical interactions and diffusible factors. In that context, it is interesting to observe that senescent cells often release factors such as cytokines or growth factors. This is known as senescence associated secretory phenotype (SASP) [5]. Recently Acosta et al have shown that the TGFβ pathway mediates paracrine senescence in SASP and that this pathway and the BMP pathway are upregulated in such senescent cells [6]. Interestingly, these two pathways are involved in tissue morphogenesis during organism development. It is therefore tempting to suggest that one of the outcomes of senescence is tissue re-organisation, achieved via cell communication, to reach new homeostasis upon cellular stress. As a matter of fact, studies of senescent cancer cells suggest so. First, senescence has been shown to act as an anti-cancer barrier, both physically and biologically in preneoplastic tissue [1]. Secondly, it has been shown to promote tumorigenesis by favouring the emergence of cancer stem-like cells (CSLCs) [7]. CSLCs are rare quiescent cells. They niche in heterogeneous tumors and have, in contrast to the bulk tumor cells but similarly to normal stem cells, the ability to self renew and to differentiate. Thus, if tissue has to be re-organised upon senescence to gain minimal homeostasis for functioning, new cells have to emerge and differentiate. This can be achieved by stimulation of CSLCs by SASP factors released from senescent cancer cells. Of note, it remains unclear why CSLCs, unlike normal stem cells, do not senesce. In relation to their role in tissue architecture, it has been described that CSLCs preferentially develop, within the tissue mass, under hypoxic conditions. Interestingly, hypoxia has been shown to inhibit mTOR, which converts quiescent cells into senescent cells [8]. If experimentally verified, hypoxia could reinforce the intrinsic resistance of CSLCs by maintaining their quiescent state, while inhibiting mTOR and geroconversion of CSLCs from quiescence to senescence.

It therefore appears, at least in pathological cancer tissue, that senescence, and SASP in particular, could play a pivotal role in tissue re-organisation upon cellular stress. As a consequence, depending on the cancer stage, i.e. to which extend tissue has to be re-organised upon cancer invasion, senescence could be pro or anti tumorigenic. As to whether this role in tissue re-organisation also occurs in non-pathological tissue remains to be investigated. Tissue re-organisation by senescence implies cellular communication through SASP factors. Therefore it will be interesting to investigate if senescence is accompanied by secretion of SASP factors in unicellular organisms. If not, this will strongly argue for a role of SASP in maintaining tissue homeostasis, via tissue re-organisation, in multicellular organisms.
